# Diagnosis of glutaric aciduria type I based on neuroradiological findings: when neonatal screening fails

**DOI:** 10.1186/s13052-025-01975-z

**Published:** 2025-05-13

**Authors:** Vincenza Gragnaniello, Andrea Puma, Daniela Gueraldi, Ignazio D’Errico, Chiara Cazzorla, Christian Loro, Elena Porcù, Leonardo Salviati, Alberto B. Burlina

**Affiliations:** 1 Division of Inherited Metabolic Diseases, Department of Women’s and Children’s Health, University Hospital of Padua, Padua, Italy; 2 Division of Inherited Metabolic Diseases, Department of Women’s and Children’s Health, University of Padua, via Orus, 2c, Padua, 35128 Italy; 3 Department of Neurosciences, University of Padua, Padua, Italy; 4 Clinical Genetics Unit, Department of Women’s and Children’s Health, University of Padua, Padua, Italy

**Keywords:** Glutaric aciduria type I, Low-excretor, Newborn screening, Glutarylcarnitine, Urinary organic acids

## Abstract

**Background:**

Glutaric aciduria type I (GA-I) is an autosomal recessive disorder affecting the metabolism of lysine, hydroxylysine, and tryptophan. Patients present in the first age of life with an irreversible motor disorder, and neuroradiological imaging can suggest the presence of the condition. Biochemically, the disorder is characterized by elevated levels of glutaric and 3-hydroxy glutaric acid in the urine and glutarylcarnitine in the blood. This latter metabolite can be detected in dried blood spots, and the condition can therefore be included in some newborn screening programs.

**Case presentation:**

We present the case of a patient affected by GA-I that was undetected by newborn screening in whom the diagnosis was clinically oriented at the age of nine months by acute neurological symptoms, represented by persistent tonic seizures, and by neuroimaging showing bilateral signal alterations in the basal ganglia. Biochemical data, including glutarylcarnitine in dried blood spots and urinary excretion of glutaric acid, were normal in the acute phase and during follow-up. Molecular analysis confirmed a diagnosis of GA-I, showing a homozygous M405V variant of the *GCDH* gene, which is common in African populations and associated with a low-excretor phenotype characteristic of the disorder.

**Conclusions:**

In conclusion, although GA-I is included in neonatal screening programs, the biochemical markers in dried blood spots can be absent. Therefore, in patients of African origin, clinicians should maintain a high degree of vigilance in the presence of suggestive clinical and neuroradiological findings, even if biochemical parameters are normal.

## Background

Glutaric aciduria type I (GA-I) is an autosomal recessive disorder caused by a deficiency of glutaryl-CoA dehydrogenase (GCDH; EC 1.3.99.7), a mitochondrial enzyme that catalyzes the oxidative decarboxylation of glutaryl-CoA to crotonyl-CoA in the catabolic pathway of lysine, hydroxylysine, and tryptophan [1–3].

In 90% of cases, infants (especially those aged between three and 36 months) develop a complex and irreversible motor disorder with predominant striatal dystonia superimposed on truncal hypotonia. These symptoms are associated with high morbidity and mortality and most often occur acutely following an acute encephalopathic crisis precipitated by intercurrent illness. However, some patients show an insidious onset without a preceding crisis. Brain magnetic resonance imaging (MRI) shows basal ganglia damage, involving bilateral damage to the putamina in particular. It is due to mitochondrial energy failure with secondary failure of Na/K ATPases and neuronal edema. Proton MR spectroscopy findings may vary with the stage of the disease. Lactate is slightly elevated during acute decompensation and in the early subacute phase; in the chronic phase, low NAA is found without lactate. Other neuroradiological findings include frontotemporal atrophy/hypoplasia, delayed myelination, and subdural and retinal hemorrhages. A total of 75% of patients present with macrocephaly at birth or throughout infancy [4–6].

The biochemical diagnosis of GA-I is based on detection of glutaric acid (GA) and 3-hydroxy glutaric acid (3-HGA) excreted in the urine and elevated levels of glutarylcarnitine (C5DC) in the blood. Two biochemical phenotypes exist amongst GA-I patients. High excretors (HE) are characterized by large quantities of GA (> 100 mmol/mol creatinine) and 3-HGA in the urine. Conversely, other patients have low/absent GA excretion (< 100 mmol/mol creatinine) and only slightly elevated 3-HGA excretion and may also have normal glutarylcarnitine levels. These individuals have been termed low excretors (LE) and represent up to 40% of patients. As a result of their less marked biochemical abnormalities, cases of LE may be difficult to identify. Diagnosis is confirmed by mutation analysis [1–3, 7].

Because C5DC can be detected by tandem mass spectrometry (MS/MS) in dried blood spots (DBS), GA-I has been increasingly included in some national newborn screening (NBS) programs, including the Recommended Uniform Screening Panel in the United States and several European countries [8, 9]. However, it should be borne in mind that newborn screening can potentially fail to identify all low excretor patients, because the biochemical abnormalities can be mild or absent.

In Italy, a nationwide newborn screening program for inborn errors of metabolism, including GA-I, was introduced by law between 2016 and 2017 (Law 167/2016). Recently, Ruoppolo et al. reported the results of NBS on 806,770 newborns screened between January 2019 and December 2020. Five neonates affected by GA-I (incidence 1:161,354) and two maternal cases were identified. False negative results were not reported at that time [10].

Here, we report the case of a patient affected by GA-I and clinically diagnosed at the age of nine months. This patient’s condition was undetected by NBS and biochemical data continued to be normal during episodes of intercurrent illness.

## Case presentation

The male patient was the third child of healthy, non-consanguineous Moroccan parents. He was born in Italy after an uneventful pregnancy and the NBS was performed correctly, with unremarkable results. The patient was asymptomatic in the first few months of life, with normal psychomotor development. At the age of nine months, he was seen in the emergency room because of a febrile upper airway infection with poor oral intake. On examination, his body weight was 9 kg (36th percentile), his length was 71 cm (36th percentile), and his head circumference was 43.5 cm (7th percentile).

**Fig. 1:**
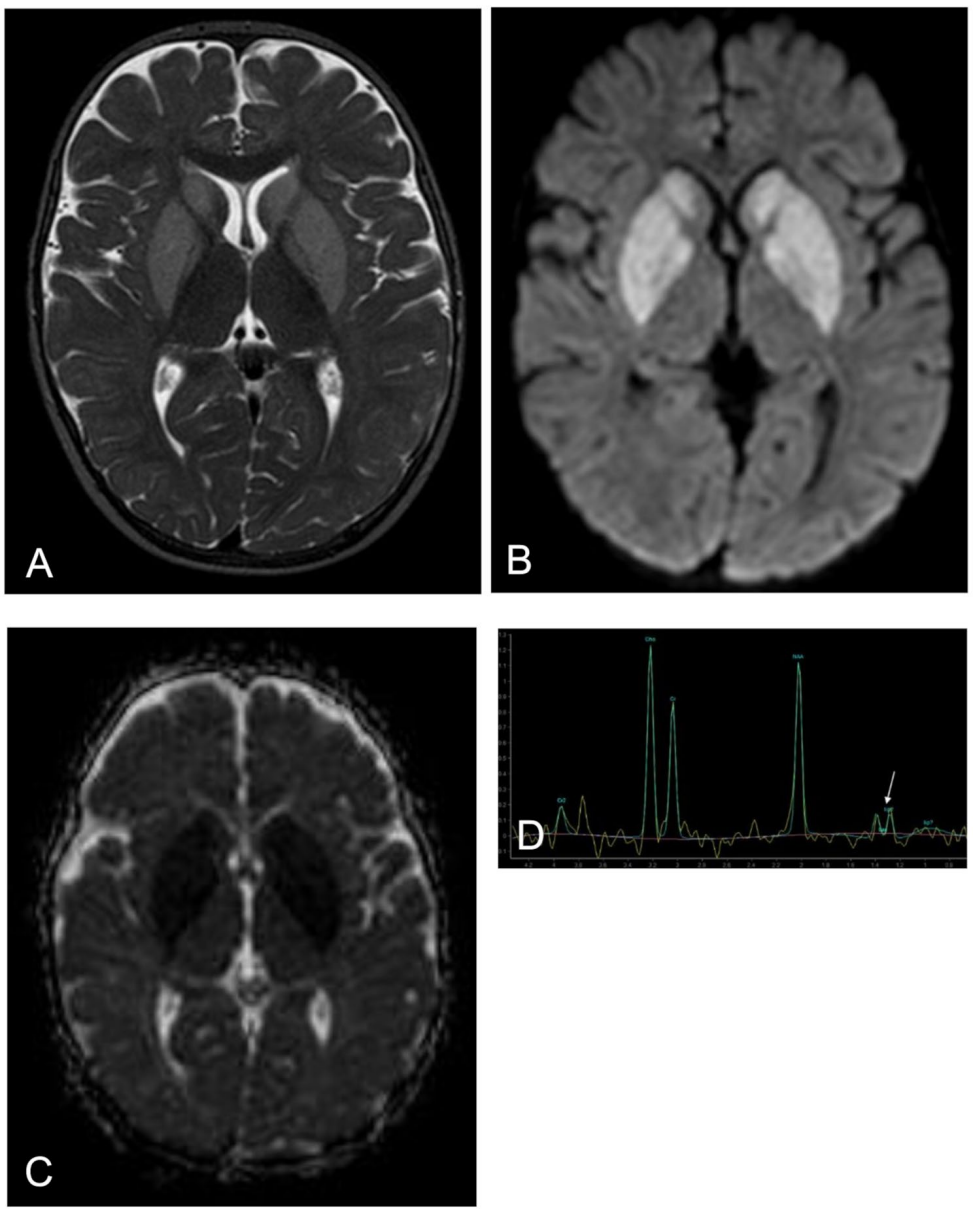
Brain MRI during the acute phase. Axial T2-weighted (**A**), diffusion-weighted (**B**) and ADC map (**C**) images show symmetrical hyperintensity and reduced diffusion in the the basal ganglia (caudate, putamen and pallidum). Proton MR spectroscopy (TE: 288 ms) shows a small lactate doublet peak at the 1.3 ppm level (**D**)

During observation in the emergency room, he presented persistent tonic seizures with progressive poor responsiveness and he was therefore transferred in the intensive care unit. Seizures were treated with levetiracetam and phenobarbital, and the patient was sedated and mechanically ventilated. Blood, urine and CSF biochemical and microbiological analysis were normal and it was noted in particular that the patient did not present acidosis or hyperammonemia. Plasma and liquor glucose and lactate were normal. A nasal swab test was positive for respiratory syncytial virus. The brain MRI showed bilateral signal alterations in the basal ganglia, affecting the caudate, putamen, and pallidum (Fig. 1). Extensive metabolic investigations were carried out at the time. Plasma amino acid levels and DBS acylcarnitine profile were normal. In particular, DBS glutarylcarnitine was 0.08 umol/L (nv < 0.25), with normal free carnitine levels (29.6 µmol/L, nv 29–42). Urinary organic acid analysis showed only slightly elevated 3-HGA levels, with absent GA. Because of the suggestive clinical and neuroradiological findings, and despite the negative NBS and the absence of typical biochemical markers (which continued during the acute phase), a diagnosis of LE GA-I was suspected. Given that urinary excretion of glutarylcarnitine (C5DC) was reported to be a specific biochemical marker that is elevated in both LE and HE GA-I patients [11, 12], tandem mass spectrometry of urine acylcarnitine was performed, but urinary C5DC in our patient was normal (1.01 µmol/mmol creatinine), with low urinary free carnitine (2.21 µmol/mmol creatinine).

High-calorie restricted-protein parenteral nutrition and intravenous carnitine supplementation (200 mg/kg per day) were initiated. After carnitine supplementation, free carnitine increased to 241.6 umol/L, but DBS C5DC remained normal (0.06–0.08 umol/l). Urinary C5DC also did not increase (1.14 umol/mmol creatinine), despite an increase in urinary carnitine (1729.64 umol/mmol creatinine). Glutarate excretion also remained undetectable. Diagnosis of GA-I was genetically confirmed, showing a compound homozygous *GCDH* genotype with pathogenic variants c.1213 A > G/p.M405V.

After 15 days, the patient was discharged from intensive care unit and transferred to our clinical unit. He presented severe truncal hypotonia, irritability, dystonic movements of limbs and neck, and dysphagia, and was dependent on tube feeding for nutrition. The lysine-restricted diet (80 mg/kg) and oral carnitine (100 mg/kg) were continued. Symptomatic treatment with trihexyphenidyl was initiated, resulting in some improvement.

**Fig. 2:**
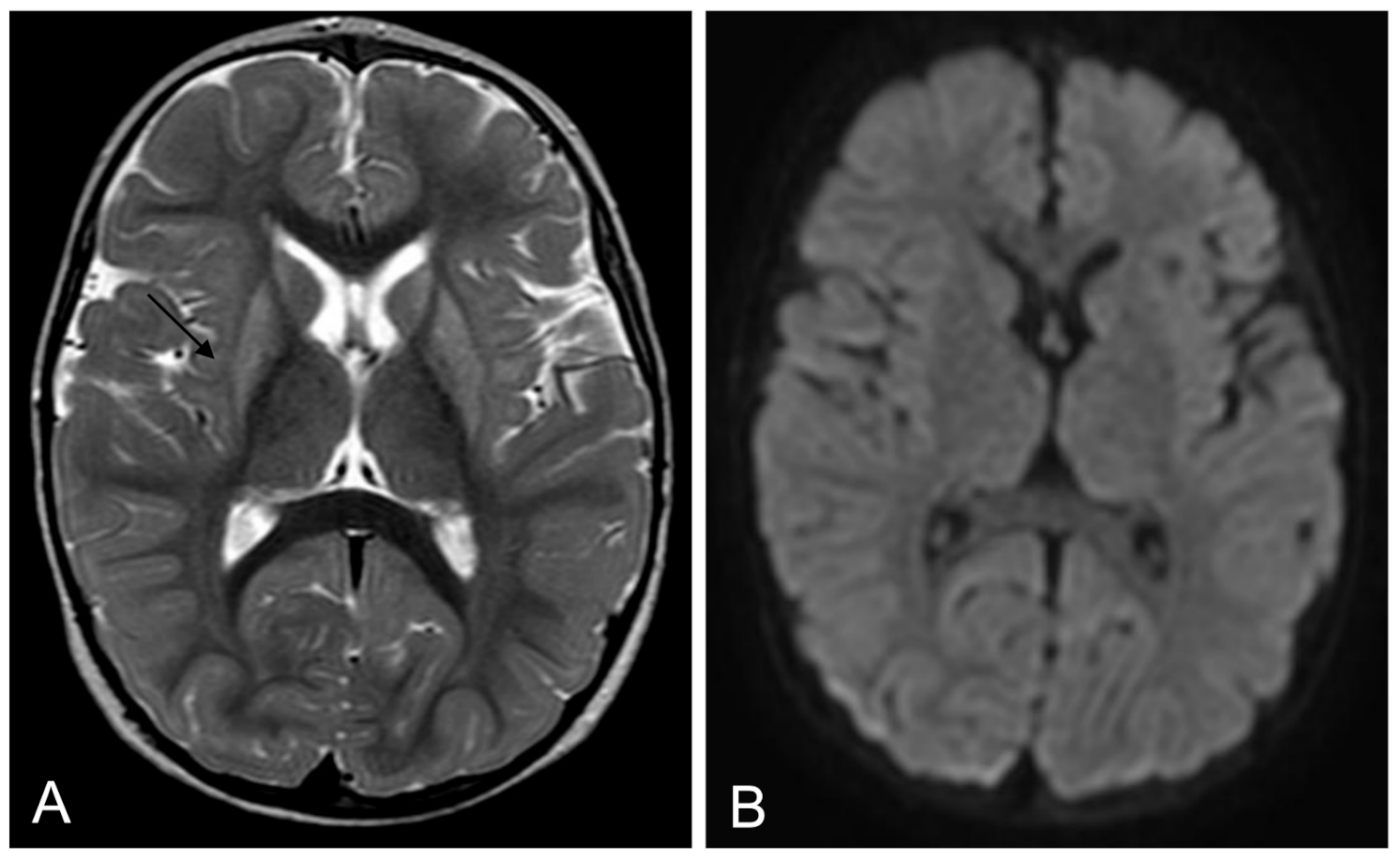
Brain MRI after 5 months. Axial T2-weighted (**A**) and diffusion-weighted (**B**) images show atrophic evolution of basal ganglia lesions and resolution of the cytotoxic edema (arrows)

At his last visit at the age of 14 months, the patient exhibited dystonic quadriplegia, with a slight improvement in hypotonia and dysphagia. He was not able to sit and was dependent on tube feeding but showed preserved good social interaction and smiling. A brain MRI showed atrophic evolution of the lenticular lesions (Fig. 2).

## Discussion and conclusions

We report on a patient affected by LE GA-I undetected by NBS and diagnosed at nine months following an acute encephalopathic crisis. During the acute phase, DBS C5DC and urinary glutaric acid were normal, and the only biochemical marker was a slight increase in urinary 3-HGA.

Diagnosis was made possible by integrating clinical and neuroradiological findings, and was confirmed only by molecular analysis of *GCDH* gene, which showed a homozygous p.M405V variant.

This is a missense variant, which alters a highly conserved *GCDH* residue [13]. It presents a high incidence in individuals of African origin, in whom the allele frequency (mean allele frequency of 0.300%) is about six times higher compared to the general population (0.053%). This variant is associated with predicted GCDH activity in a M405V homozygote that is between 8% and 35% of control values, compatible with an LE phenotype [11].

This variant was first described by Korman et al. in a heterozygous S119L/M405V Jewish patient with a typical course of symptomatic GA-I. The patient had not undergone NBS and, like our patient, he was diagnosed at the age of eight months following acute presentation with seizures and dystonic movements during an intercurrent illness. Biochemically, he presented no GA excretion, only mildly abnormal excretion of 3-HGA, and only minimally elevated DBS C5DC but increased urinary excretion of C5DC. A brain MRI showed signal abnormalities in the caudate nucleus, putamen and globus pallidus, but the typical Sylvian fissure and pretemporal abnormalities were lacking [13].

Subsequently, Schillaci et al. reported on nine cases of LE GA-I patients, all sharing the M405V allele in compound heterozygous forms, of whom four had undergone NBS and had been identified as negative. GCDH activity was assayed for six of the nine patients and varied from 4 to 25% of the control mean [11].

A few other cases of GA-I have been reported as being missed on NBS and diagnosed after the onset of symptoms [14–18]. In some cases, the DBS from the NBS were retrospectively assayed and identified as negative [15, 16]. All had an LE phenotype and, when assessed, high residual enzyme activity (10-25%) [16]. The clinical onset, between five and 28 months, was in the form of acute encephalopathic crisis [15–18] or a more insidious scenario [14, 17, 18]. In most cases, as in our patient, the macrocephaly characteristic of GA-I was absent [16, 17]. Moreover, all patients exhibited involvement of the basal ganglia [15–17], but no other typical neuroradiological findings of GA-I, such as the widening of the sulci and Sylvian fissure, dilation of the lateral ventricles [15], frontotemporal atrophy [16], and abnormal white matter signals [19]. At the time of diagnosis, all patients showed at least slightly elevated urinary 3-HGA [15–18], reinforcing the importance of this biomarker for the diagnosis of GA-I [11, 20]. Urinary GA was normal [15, 17, 18], as was DBS C5DC, even with carnitine supplementation [17]. Only a few patients presented with increased urinary C5DC [7, 18], a specific biochemical marker [11, 12] that was normal in other cases, as it was in our patient.

Given the significant residual enzymatic activity in the cases described above with the LE phenotype [11], it is conceivable that this could be responsible for the partial neuroradiological anomalies and the mild biochemical phenotype. However, both are equally likely to manifest severe complications of GA-I and, therefore, LE should not be mistaken for a “mild” disease variant [21].

The diagnosis was confirmed by mutation analysis of the *GCDH* gene. In addition to M405V [16–18], the most frequent variants were R227P [15] and V400M [16], associated with an LE phenotype.

Given the prevalence of these variants in African populations and recent migratory flows, a high degree of vigilance should be maintained in patients of African origin, even if they have undergone NBS in European countries with negative results.

The exact number of missed patients is not known, as NBS programs often lack a tracking system. In the German NBS program, three patients have been missed in about five million newborns. The overall sensitivity of C5DC screening in a mixed population is about 95% [17], with a discrepancy between patients with the HE (100%) and LE (84%) phenotypes [8]. In our experience, after ten years of newborn screening, four patients were identified and one patient was missed, demonstrating a sensitivity of 80%.

To conclude, the case presented here demonstrates that newborn screening for GA-I using tandem mass spectrometry may miss some affected individuals, particularly those with an LE phenotype, because patients can exhibit a mild biochemical phenotype with normal DBS C5DC concentrations. These patients might not come to clinical attention until they experience an acute intercurrent episode of encephalopathic crisis with severe irreversible neurological sequelae. Biochemical investigations do not rule the condition out and diagnosis can only be made through molecular analysis. A high degree of awareness by clinicians is important in the presence of suggestive clinical and neuroradiological findings and should also be maintained in patients with normal NBS results and where typical biochemical markers, such as DBS C5DC and urinary glutaric acid, are absent.

## Data Availability

Data available on request due to privacy/ethical restrictions.

## Abbreviations

3-HGA: 3-hydroxy glutaric acid
C5DC: Glutarylcarnitine
DBS: Dried blood spot
GA: Glutaric acid
GA-I: Glutaric aciduria type I
HE: High excretors
LE: Low excretors
MRI: Magnetic resonance imaging
MS/MS: Tandem mass spectrometry
NBS: Newborn screening
